# Insights and challenges of insecticide resistance modelling in malaria vectors: a review

**DOI:** 10.1186/s13071-024-06237-1

**Published:** 2024-04-03

**Authors:** Eric Ali Ibrahim, Mark Wamalwa, John Odindi, Henri Edouard Zefack Tonnang

**Affiliations:** 1 International Centre of Insect Physiology and Ecology (Icipe), PO box 30772, Nairobi, Kenya; 2 School of Agricultural, Earth, and Environmental Sciences, University of KwaZulu-Natal, Pietermaritzburg, 3209 South Africa

**Keywords:** Spatial, Bayesian geostatistical, Temporal, Spatio-temporal, Ecological principles, Processes

## Abstract

**Background:**

Malaria is one of the most devastating tropical diseases, resulting in loss of lives each year, especially in children under the age of 5 years. Malaria burden, related deaths and stall in the progress against malaria transmission is evident, particularly in countries that have moderate or high malaria transmission. Hence, mitigating malaria spread requires information on the distribution of vectors and the drivers of insecticide resistance (IR). However, owing to the impracticality in establishing the critical need for real-world information at every location, modelling provides an informed best guess for such information. Therefore, this review examines the various methodologies used to model spatial, temporal and spatio-temporal patterns of IR within populations of malaria vectors, incorporating pest-biology parameters, adopted ecological principles, and the associated modelling challenges.

**Methods:**

The review focused on the period ending March 2023 without imposing restrictions on the initial year of publication, and included articles sourced from PubMed, Web of Science, and Scopus. It was also limited to publications that deal with modelling of IR distribution across spatial and temporal dimensions and excluded articles solely focusing on insecticide susceptibility tests or articles not published in English. After rigorous selection, 33 articles met the review's elibility criteria and were subjected to full-text screening.

**Results:**

Results show the popularity of Bayesian geostatistical approaches, and logistic and static models, with limited adoption of dynamic modelling approaches for spatial and temporal IR modelling. Furthermore, our review identifies the availability of surveillance data and scarcity of comprehensive information on the potential drivers of IR as major impediments to developing holistic models of IR evolution.

**Conclusions:**

The review notes that incorporating pest-biology parameters, and ecological principles into IR models, in tandem with fundamental ecological concepts, potentially offers crucial insights into the evolution of IR. The results extend our knowledge of IR models that provide potentially accurate results, which can be translated into policy recommendations to combat the challenge of IR in malaria control.

**Graphical Abstract:**

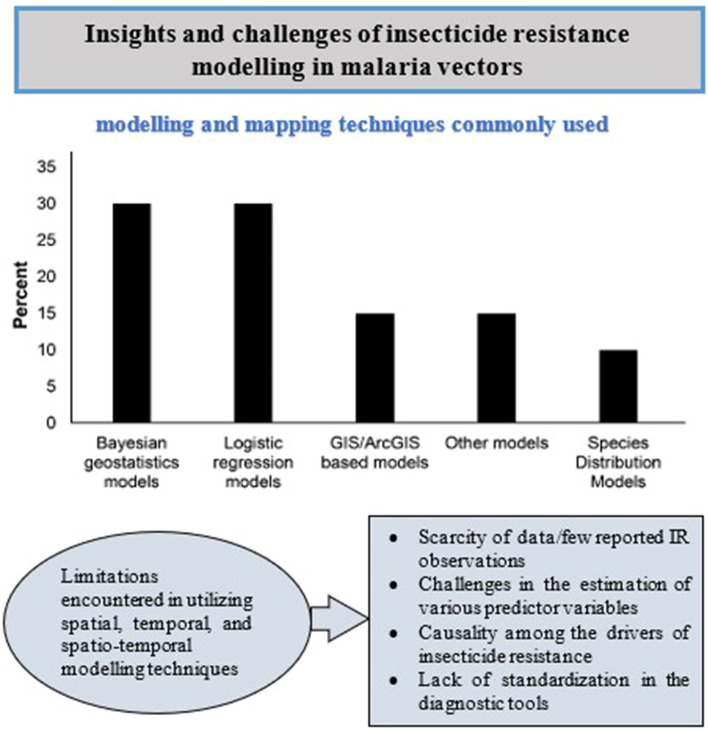

**Supplementary Information:**

The online version contains supplementary material available at 10.1186/s13071-024-06237-1.

## Background

Global malaria cases have gradually increased, with records showing an estimated rise of 232, 247, 245 and 261 million cases in 2019, 2020, 2021 and 2022, respectively [1, 2]. Despite the different control strategies for malaria prevention, the estimated number of deaths in the same period were 568,000, 619,000, 625,000 and 710,000 in 2019, 2020, 2021 and 2022, respectively [1, 2]. Moreover, trends reveal a stall in the progress against malaria transmission in countries that have moderate or high malaria transmission, due to intrinsic obstacles [e.g. insecticide resistance (IR)] encountered while using the available malaria control tools and strategies [3, 4].

In Africa, National Malaria Control Programmes (NMCPs) have developed strategic plans on the basis of World Health Organization (WHO) recommendations, which guide the control and prevention of malaria transmission. The strategic plans involve a combination of clinical treatment of the disease with vector surveillance and control, most often using insecticides [5, 6]. However, resistance to these chemical interventions is now widespread in vector populations, and in particular the anopheline species, which transmit the malaria parasite [6]. For example, a numbers of studies (e.g. [3, 4, 6, 7]) have reported IR to the commonly used insecticide classes (pyrethroids, carbamates, organophosphates and organochlorine) in most African countries. Typically, resistant mosquitoes survive the exposure to standard doses of the insecticides used in indoor residual house spraying (IRS) and long-lasting insecticidal nets (LLINs), hence threatening the success of the fight against malaria transmission [8, 9]. There are four major underlying mechanisms of IR that have been widely documented, namely, target-site mutation, metabolic, behavioural and cuticular (penetration) resistance [10–12].

Target-site resistance is caused by mutations at the site of action of an insecticide, thereby reducing or preventing the insecticide binding affinity [3, 6, 13, 14]. Conversely, metabolic resistance entails increased detoxification of insecticides by the vector through overexpression or conformational changes of the enzymes that can metabolize, sequester and excrete the insecticide [4, 15]. Behavioural resistance involves any modification in the insect’s behaviour that helps it to avoid exposure to insecticides. In malaria vectors, this is commonly observed as a change in biting patterns, for example, biting earlier and outdoors, thus avoiding any LLINs or IRS [4, 16]. Cuticular resistance is seen when the insect’s cuticle thickens, reducing uptake of insecticide by limiting or preventing the absorption or penetration of insecticide [3, 4].

Monitoring IR within malaria vector populations requires comprehensive surveillance to determine where it is emerging, to what degree and how it is spreading in space and time [11]. Extensive, albeit spatially restricted, studies have been carried out to establish the emergence and dissemination of IR in contemporary African malaria vector populations and its impact on the efficacy of control strategies adopted [17, 18]. However, given the impracticality of establishing this critically needed information at every location, there is a growing reliance on modelling to make predictions in situations in which data are absent or limited. In addition, several countries have established longitudinal monitoring in sentinel sites, enabling detection of temporal changes in the prevalence of resistance [19]. With increasing need to establish the trend of IR, Hancock et al. [11] point out the increasing necessity of analysing the spatial–temporal variation of IR across multiple countries.

It is therefore imperative to conduct a review of the modelling techniques employed thus far, explore avenues to bolster models’ robustness and address the challenges encountered. Additionally, attaining a comprehensive grasp of the drivers that contribute to the dissemination of IR among malaria vectors across spatial and temporal dimensions is of paramount importance. This understanding is critical, as IR driving factors significantly contribute to the establishment of a robust foundation for the resultant model [11]. Anchored on the inception and propagation of IR, it is imperative to consider the role played by ecological processes and principles. These elements serve as orchestrators of crucial cellular processes, physiological activities, insect movements and the intricate mechanisms governing metabolic detoxification [20, 21]. The balance between heat utilization and storage by the insects based on internal and external environmental conditions, such as temperature, is a potential influencer of insects’ responses to stressors [22]. Consequentially, the heightened production of enzymes to detoxify insecticides leads to depletion of heat reserves in mosquitoes [23].

Given the interrelation of heat and temperature with IR among malaria vectors, ecological principles such as thermodynamics assume a potentially crucial role in modelling IR. Specifically, the second principle of thermodynamics, which posits that processes involving the transfer or conversion of energy are inherently irreversible and tend to result in increased disorder (entropy), becomes particularly relevant. It is important to note that entropy in the universe is perpetually non-negative, a fundamental concept that could hold valuable insights for understanding and modelling IR dynamics [24]. Moreover, the migration of resistant vectors to new areas holds the potential to significantly contribute to the propagation of IR [25]. Such dissemination signifies an irreversible flow, consequently leading to an escalation in entropy. Although literature hints at a conceivable connection between thermodynamic principles and the occurrence/spread of IR, it remains unclear whether such considerations are factored into existing models.

Hence, this paper presents a review of the existing studies aimed at establishing the distribution patterns of IR within field vector populations. This review explored the modelling methodologies employed to elucidate the spatial and temporal occurrence and propagation of IR in field vector populations. Specifically, we addressed the following research inquiries:

1. Which modelling techniques are commonly employed to elucidate the spatial, temporal and spatio-temporal trends of IR in malaria vectors?
2. Which IR driving factors are utilized as inputs to inform the development of spatial, temporal and spatio-temporal models?
3. What are the limitations encountered in utilizing IR spatial, temporal and spatio-temporal modelling techniques?
4. To what extent have ecological principles, including thermodynamics, been incorporated into the study of insects and their development of IR?

Through these insights, we anticipate that future advancements and refinements in modelling techniques can be achieved, thus contributing to the overall enhancement of our comprehension of IR distribution and dynamics.

## Methods

### Literature search, inclusion and exclusion criteria

To select the articles for review, we followed the Preferred Reporting Items for Systematic reviews and Meta-Analyses (PRISMA) procedures, which delineate different phases of a systematic review that includes identification, screening, eligibility and inclusion criteria [26]. We conducted literature search in three databases, that is, PubMed, Scopus and Web of Science, with the search based on various themes, namely: (a) insecticide resistance, (b) malaria vectors, (c) modelling and (d) ecological principle in IR diffusion and thermodynamics. For each theme, we employed specific keywords, including “insecticide resistance”, “malaria”, “vector*”, “model”, “thermodynamics*”, “ecological principle” and “diffusion”. Combining these keywords, we customized our search strategy for each database to maximize specificity. We did not impose restrictions on the initial publication year of the articles, with our search extending until March 2023. Table 1 presents a summary of the keywords used.

**Table 1:** Keywords used in database searches in systematic review

Theme	Keywords
Insecticide resistance	“insecticide resistance”
Malaria vectors	“malaria” AND “vector*”
Modelling	“model*”
Thermodynamics	“Thermodynamics”
Ecological principle	“Ecological principle”
Diffusion	“Diffusion”

^*^Asterisk used in a search query

### Eligibility criteria

This review was specifically confined to studies that addressed spatial, temporal and spatio-temporal modelling of IR in malaria vector populations, with a particular emphasis on the incorporation of thermodynamics, diffusion and ecological principles into the models. Therefore, the eligibility criteria involved selection of articles that exclusively explored IR in malaria vectors, with a specific focus on spatial and spatio-temporal IR distribution modelling. We excluded articles related to IR tests in malaria vectors and those that employed alternative IR modelling approaches beyond spatial, temporal and spatio-temporal modelling. Additionally, we limited our selection to articles published in English. A detailed eligibility criteria is presented in Table 2.

**Table 2:** Eligibility criteria

	Inclusion criteria	Exclusion criteria
Insecticide resistance	Insecticide resistance	Only testing for insecticide resistance
Vectors	Malaria vectors and their spatial distribution	Other vectors
Modelling	Spatial and spatio-temporal modelling	Modelling techniques not relating to spatial and spatio-temporal modelling of IR in malaria vectors
Thermodynamics	Thermodynamics in the context of insects	Thermodynamics in context not related to insects
Ecological principles	Diffusion process	Diffusion process related to insecticide resistance
Language	English	Other languages

### Screening and selection of articles

During the search process, the articles were subjected to a three-phase screening procedure in accordance with the PRISMA flow diagram (see Page et al. [26]). The initial phase involved filtering articles on the basis of keywords to identify those relevant to our study. In the second phase, we manually screened the selected articles by reviewing their titles and abstracts, eliminating those that did not meet the eligibility criteria. Subsequently, we consolidated articles from each database and conducted a comprehensive assessment of the full text for each article after removing duplicates.

### Risk of bias assessment

We conducted a narrative synthesis of all the articles included in the review and presented the results in a tabular format. To evaluate biases pertinent to our review, we followed the guidelines outlined in the Systematic Review Centre for Laboratory Animal Experimentation (SYRCLE) guide [27].

## Results

### Search results

**Fig. 1:**
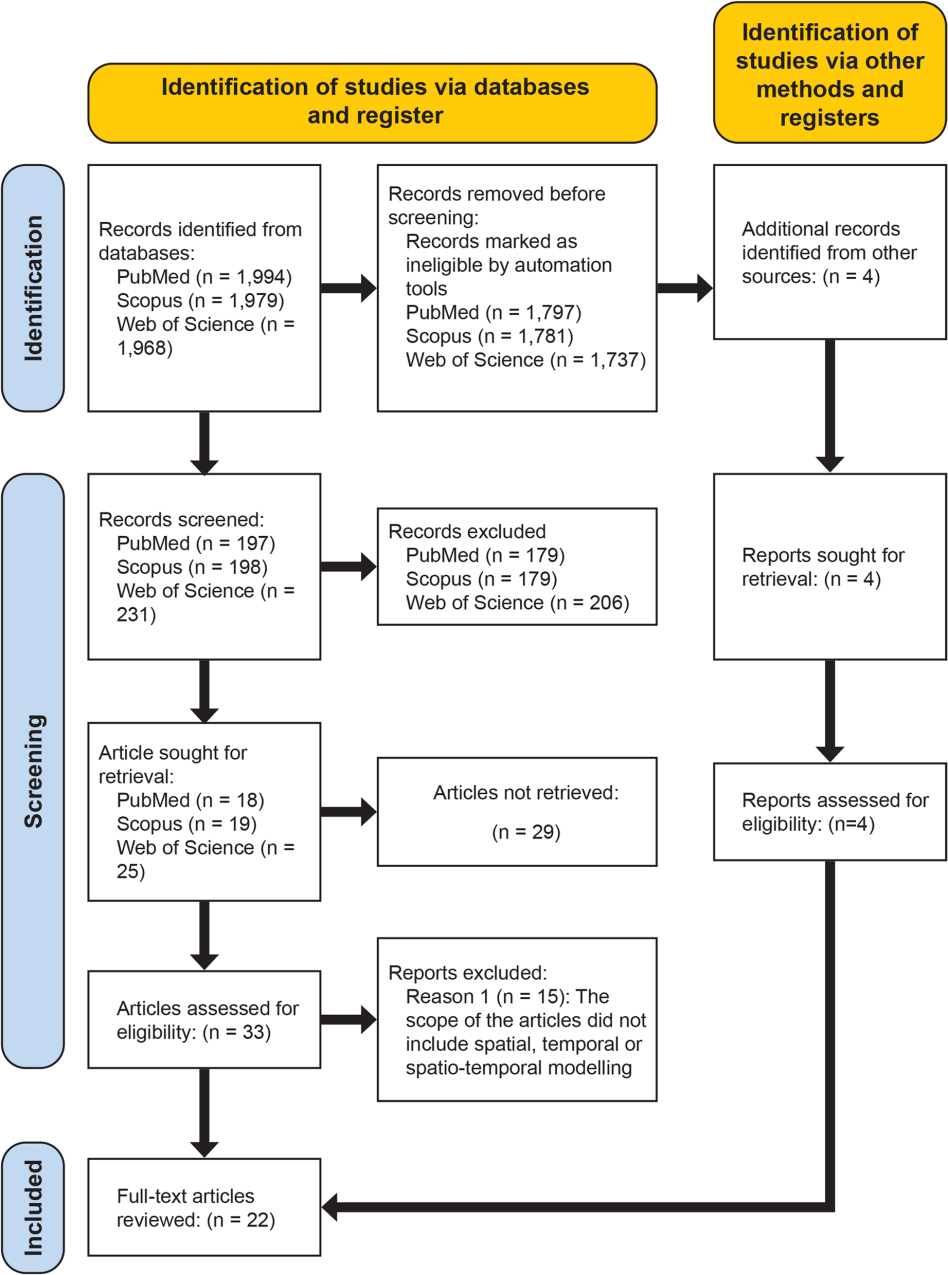
PRISMA flow chart of the search phases and the number of records retrieved

In the databases chosen for the study (i.e. PubMed, Scopus and Web of Science databases), we initially retrieved a total of 1994, 1979 and 1968 articles, respectively. After removing duplicate entries and conducting a rigorous screening process, we identified 18 articles that met our criteria for full review. Additionally, we included four (4) articles identified through citation searches for further evaluation. When conducting a combined search using all keywords we obtained zero results, hence we excluded the term ‘thermodynamics’ from the combined search. Subsequently, we conducted a separate search specifically for ‘thermodynamics’, resulting in the retrieval of five articles. Figure 1 illustrates the search results.

### Summary of the reviewed articles

The summary details of the articles [10–12, 28, 29, 33, 45, 52–66]  included in the final review are presented in Additional file 1: Table S1. The articles’ summary includes the aim, the methodology adopted and findings of the studies (Additional file 2).

### Risk of bias assessment

Selection bias, encompassing sequence generation and allocation concealment, did not pertain to this systematic review. Furthermore, none of the reviewed articles included blinding of participants or investigators, excluding blinding (performance and detection bias) from the review. Additionally, the risk of bias due to attrition was not applicable to our review. Importantly, reporting risks were consistently low across all 22 studies, as they diligently adhered to the pre-specified methodology in providing detailed and coherent findings.

### Equity analysis

Equity analysis showed that the majority (65.97%) of the institutions affiliated with the authors were located in countries that are not hyperendemic. The remaining institutions (34.03%) were situated in hyperendemic countries.

### Spatial and spatio-temporal modelling of insecticide resistance in mosquito vectors

**Fig. 2:**
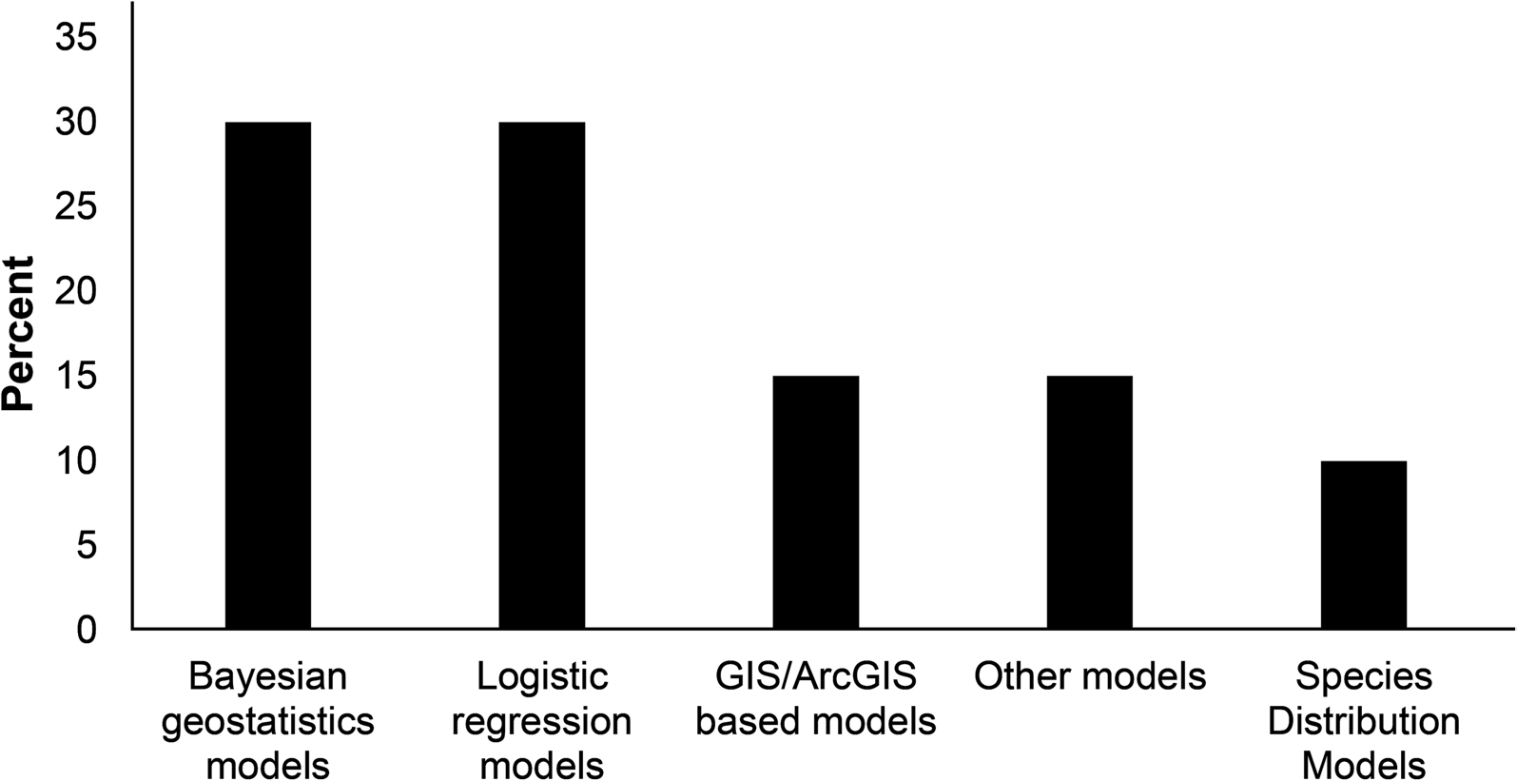
Models used to determine IR trends and distribution of malaria vectors. GIS implies geographic information system (GIS)

The reviewed articles used different mapping and modelling techniques to elucidate IR trends in malaria vectors. These approaches encompassed mapping occurrence of IR with techniques such as geographic information system (GIS), Bayesian geostatistical models, generalized linear models (GLMs) and generalized additive models (GAMs). Species distributions models were used to map vectors’ distribution but not IR in vectors’ population. Notably, none of the studies employed dynamic models to investigate the spatio-temporal distribution of IR in malaria vectors across both space and time. In addition, several modelling approaches involved splitting the data into training and test sets. This process involves training the model with the training sets and subsequently evaluating its performance using the test set. Figure 2 provides a summary of the modelling techniques employed.

### Drivers for the models on spatial and spatial–temporal trends of insecticide resistance

The studies employed various factors as drivers for informing the spatial modelling of IR in malaria vectors. These encompassed, among others, climatic, sociological and environmental conditions, as well as the extensive use of insecticides in both agricultural and public health sectors. Some of the reviewed articles also incorporated lagged temporal variables, positing their influence on IR patterns. Nevertheless, other studies chose not to lag temporal variables, suggesting that such lagged variables might not significantly impact IR distribution within the model. Biological variables were also considered in some studies. Notably, very few studies explored the potential influence of vector dispersal and thermodynamics on the spread of IR across space and time. Table 3 provides a summary of the diverse drivers employed in these analyses.

**Table 3:** Summary of potential driver of insecticide resistance in malaria vector populations

	Drivers of insecticide resistance
1	Rainfall
2	Temperature
3	Relative humidity
4	Precipitation
5	Elevation
6	Vegetation index
7	Wind speed and direction
8	Land-use and land-cover
9	Hydrology
10	Solar radiation
11	Surface wetness
12	Minimum surface wetness
13	Non-food crop area
14	Insecticide coverage
15	Processes leading to pesticides’ fate in the environment
16	Distance from water bodies
17	Crops and livestock farming
18	Oil spillage
19	Human population distribution
20	Drainage

### Challenges encountered in modelling insecticide resistance among vectors

The reviewed articles underscore multiple challenges encountered in modelling IR among the vectors. These challenges encompass a spectrum of issues, which include the scarcity of observations in field populations in certain regions and the lack of data on potential drivers. A summary of these challenges is provided in Table 4.

**Table 4:** Challenges faced in modelling insecticide resistance in space and time

Challenges encountered
(a) Scarcity of data/few observations
Scarcity and heterogeneous distribution of IR data in some regions
In another context, few observations resulted in higher precision errors while using Bayesian geostatistical models to model the distribution of vectors in Mali
(b) Challenges in the estimation of various predictor variables
There were challenges in the estimation of various predictor variables such as quantities of insecticides used in agriculture and where they are used
(c) Causality among the drivers of insecticide resistance
Establishing causality among the drivers of insecticide resistance in malaria vector populations could be explored further because the variables interact. In addition, causal variables used to develop various models may not have been exhaustive, hence use of additional potential insecticide resistance drivers may result in more robust models
(d) Lack of standardization in the diagnostic tools
Estimating insecticide resistance in un-sampled locations is hampered by a lack of standardization in the diagnostic tools used and by a lack of data in several regions for both resistance phenotypes and genotypes

### Thermodynamics

Following our review, no study investigated the role of thermodynamics on IR diffusion over space and time. However, a few studies discussed thermodynamics in the context of insects, as presented Table 5.

**Table 5:** Thermodynamics and insects activities

Author	Aims	Summary of the article
[47]	To determine how thermodynamics constrains the evolution of growth rates of insect population	Population dynamics of insects were altered by the adaptation to temperatures. In addition, diverse physiological and biochemical adaptations allow ectotherms to survive and reproduce in different temperature regimes
[48]	To determine the extent to which thermodynamics of biological rates constrains the thermal adaptation of developing ectotherms	Using biophysical Sharpe–School field model, the study explained the dependence between temperature and body size in ectotherms, and predicted the temperature tolerance limits in developing ectotherms and patterns of thermal adaptation among and within the species. On the basis of the study findings, the enzyme activity–stability trade-off is the most important thermodynamic constraint and limits the viable development of majority of ectotherms to a relatively small thermal tolerance range
[49]	To understand the thermal sense of blood-sucking insects and why physics matters in such context	The loss of heat by endotherms to the environment happens if the environment is colder. Such energy is absorbed by other ectotherms, and this can make them increase their temperature, which in turn can activate specialized molecular receptors and generate a nervous signal. Insects, therefore, need to deal with the two variants of thermal information, which are fluctuations of temperature and heat exchange
[50]	To understand insect thermodynamics	The study pointed out that the accuracy of inferences on how organisms respond to thermal perturbations in their environments depends on two parameters, namely, the temperature of an organism under prevailing microclimatic conditions, and secondly, the organism’s performance at that temperature
[51]	To understand the thermodynamic properties of insect swarms	Insect swarms are well represented as van der Waals gases, and the possibility of thermodynamic cycling is attributed to the swarms consisting of several overlapping sublayers

## Discussion

This review shows that diverse ranges of field studies have focussed on confirming the existence of IR in malaria vector populations within specific regions. Furthermore, numerous studies have delved into susceptibility tests and few on modelling and mapping of the spatial distribution of IR among malaria vectors. The growing emphasis on mapping and modelling the spatial distribution of IR in malaria vector populations can be attributed to the escalating demand for effective malaria control strategies and the management of IR in these vectors [6]. The studies under review have employed various methodologies for modelling and mapping IR distribution. These approaches encompass Bayesian geostatistical models, generalized linear models (GLMs) which generally extends the traditional linear regression model [67], generalized additive models (GAMs) which are extensions of GLMs [68], and geographic information systems (GIS). The outputs generated by these models hold considerable significance in shaping strategies for IR management and control [11, 28, 29].

Nevertheless, the resulting models are often static, meaning that once these studies formulate the models, they tend to use them without subsequent updates. Furthermore, there is a noticeable underutilization of dynamic modelling approaches in modelling IR distribution. Dynamic modelling approaches offer the advantage of adaptability, allowing the model to be continuously updated as new data become available. This is particularly valuable when modelling phenomena are characterized by temporal dynamics. Regardless of the specific mechanisms involved, whether they are target-site mutations, metabolic modifications, behavioural adaptations or changes in the mosquito cuticle, they all contribute to the development of IR in mosquito populations. This result underscores the potential of dynamic modelling as a holistic approach to grasp the evolution of IR. Yet our review of the literature indicates a significant lack of dynamic modelling applications in studies on vectors, despite their proven efficacy in predicting the spread of certain insects, such as *Tuta absoluta,* with notable accuracy [30].

Additionally, a notable observation is that various models featured in the reviewed articles primarily focus on spatial aspects of IR distribution within vectors at discrete timepoints, often neglecting temporal trends. Many studies employ data-driven modelling approaches; hence, the robustness of these models is heavily contingent on the quality and quantity of the data utilized. Nonetheless, a significant challenge encountered in the development of models for IR distribution is the scarcity of data, particularly in regions where the status of IR distribution remains unknown. In this context, a comprehensive comprehension of spatial variations, temporal patterns and future analyses necessitates robust data coverage across both spatial and temporal dimensions, ideally including standardized measures of IR [31]. The idea presented in Hancock et al. [11] that tackles the challenge of data scarcity involves creating models informed by available data, thereby facilitating predictions in regions with insufficient IR data.

The IR datasets utilized in the reviewed studies were derived from a mix of sources, including primary data collection and secondary data from existing databases, such as the vector atlas database (https://vectoratlas.icipe.org/) [32]. These databases, vital for providing essential IR data, compile their contents from a range of sources, including published reports, peer-reviewed scientific journals, governmental and non-governmental organizations and research institutions. Such data play a crucial role in supporting efforts to combat malaria. Nonetheless, collecting these data are challenging. Furthermore, inconsistencies in data reporting and a lack of standardized methodologies present significant obstacles to utilizing these data effectively. Additional limitations include the absence of bioassay studies or surveillance in various locations and the reluctance or failure of data holders to share their findings, leading to gaps in data availability. This scarcity of data, coupled with the challenges of data collection and standardization, hampers the comprehensive understanding and management of IR.

In exploring alternative strategies in situations of limited data availability, one promising avenue is the utilization of mechanistic modelling approaches. These approaches hold considerable potential as they do not heavily rely on extensive data inputs compared with data-driven models. Mechanistic models are premised on an understanding of the underlying biological, ecological or physical processes driving the phenomenon of interest. Mechanistic models often involve the development of mathematical equations or simulations that capture the fundamental mechanisms governing the system. These models can provide valuable insights into the dynamics of IR, even when there are a scarcity of empirical data. By incorporating knowledge about the biology, behaviour and genetics of the vector species, as well as the mechanisms of insecticide action, mechanistic models can help simulate and predict how IR may evolve and spread over time. While data-driven models rely heavily on available observations, mechanistic models offer an advantage in situations in which empirical data are limited or unavailable. They allow researchers to make informed predictions on the basis of a fundamental understanding of the underlying processes. However, it is important to note that mechanistic models require a strong theoretical foundation and a thorough grasp of the relevant biological and ecological factors, making them a valuable tool in addressing data scarcity challenges in IR modelling.

The drivers used to inform IR models have an implication on the robustness of the resulting models [11]. Commonly used drivers of IR include climatic, environmental, topographic and biological factors [11, 28, 33, 34]. In addition, the spatial distribution of malaria vectors is highly influenced by human population density, irrigated natural/crop landscapes, areas with water pools and dense scrublands [35]. The vector occurrence and abundance also vary considerably in space and time, depending on seasonal conditions, presence of interventions, and smaller scale heterogeneity such as proximity to productive larval sites. The reviewed articles note that the list of drivers may not be exhaustive, and therefore inclusion of additional drivers can potentially result in a more robust model [11]. Hence, this indicates the need to explore additional potential drivers of IR in malaria vector populations. In addition, the trend shows use of a large number of variables to inform IR models. This increases possibilities of collinearity among the variables.

In modelling, the process of model validation is crucial for ensuring the accuracy, reliability and trustworthiness of the outputs [36]. Model validation also serves to determine whether a model is overfitting; that is, it performs well on the training data but fails to generalize effectively to unseen data. In the studies reviewed, the use of a test set (unseen data) is a common practice for model validation. This approach involves setting aside a portion of the data as a test set to evaluate the model’s performance after it has been trained on the training set. Additionally, other model validation techniques, such as cross-validation [11], are utilized. Cross-validation is a resampling technique used to evaluate a model’s performance on a limited data sample [37]. This method involves dividing the data sample into a specified number of partitions, denoted by the parameter k, to systematically validate the model across different subsets of the data. On the basis of the findings of this review, it is evident that the existing models for IR in vector populations are yet to incorporate the principles of thermodynamics and diffusion principles. Nevertheless, theoretical advancements have recognized that the physiological processes of malaria vectors and other ectothermic organisms, including the metabolism of insecticides, are profoundly influenced by temperature and environmental conditions. Moreover, temperature fluctuations play a significant role in determining the efficacy of insecticides. Specifically, elevated temperatures tend to reduce the effectiveness of insecticides, as they accelerate the rate of metabolism and alter the underlying biochemical processes [33, 38, 39].

Energy plays a crucial role in various physiological processes within an insect, encompassing movement, respiration, immunity and metabolism [40]. Insecticides exert their effects by disrupting the energy balance within the vectors’ nervous systems, ultimately leading to their paralysis and demise. In response, insecticide-resistant insects employ various mechanisms, such as enhanced detoxification of the insecticide or mutations in the target site, which reduce the binding affinity for the insecticide. These mechanisms involve energy-intensive processes, demanding the insect to expend more energy. This highlights the relevance of thermodynamic principles in the context of understanding the spread of IR over time and space. Another thermodynamic concept pertinent to IR is entropy. As the prevalence of resistance alleles in populations increases, entropy also rises [41]. Strikingly, among the reviewed articles, none have incorporated thermodynamic principles into the modelling of spatial IR distribution. This underscores the potential for further exploration in this area, as the integration of thermodynamics could yield valuable insights into the dynamics of IR within vector populations. Various mathematical expressions and probability distributions play a crucial role in estimating principles, including those related to thermodynamics. The Boltzmann distribution, for instance, is utilized in calculating entropy [42]. This distribution can also be applied to quantify thermodynamic properties, thereby allowing for their integration as a covariate in insecticide resistance (IR) models. The integration of thermodynamics into modelling has been demonstrated in other contexts, notably in describing the relationship between insect development and temperature [38, 42–44]. Incorporating thermodynamic principles into IR models could enhance our understanding of the randomness and disorder inherent in the evolution of resistance, potentially leading to more accurate predictions and insights into resistance mechanisms.

Furthermore, it is noteworthy that a significant gap exists in the consideration of migration or dispersal as potential drivers influencing the spatial distribution of IR in vectors over both space and time. The role of migration and dispersal should not be dismissed in this context, as the movement of adult mosquitoes has the capacity to significantly transform their spatial and temporal distribution. Migratory or dispersal events can introduce genetic diversity, including resistance alleles into previously unaffected regions, impacting the local dynamics of IR. Understanding the extent and patterns of vector migration and dispersal is crucial, as these factors can influence the spread of resistance, particularly in areas with varying selective pressures. However, it is important to recognize that the incorporation of migration and dispersal into models for IR distribution is a complex endeavour, requiring data and a nuanced understanding of vector behaviour and movement patterns. Therefore, this aspect represents a gap for further exploration and integration into future research efforts aimed at comprehensively modelling IR in vector populations [41–45]. Vectors have also been observed to disperse for various reasons, including searching for food, mates and oviposition and resting sites. These dispersal behaviours exert a notable influence on their spatial distribution [45, 46].

Another critical issue that is often overlooked when developing models to explain the combined spatial and temporal evolution of IR is the initial conditions in both time and space. In the context of modelling, ‘initial conditions’ refer to the starting values or states of the system under study. These conditions serve as the foundation upon which the model begins to simulate the dynamic processes over time and across space. The importance of accurate initial conditions cannot be understated. They significantly influence the trajectory and outcomes predicted by the model. Without proper initial conditions, the model may not faithfully represent the real-world scenario, potentially leading to inaccurate or unreliable predictions. For example, when modelling IR dynamics in a specific region, it is crucial to know the initial prevalence and distribution of resistance alleles among the vector population at the beginning of the study period. Likewise, the starting environmental conditions and selective pressures are equally vital. Failure to account for these initial conditions can result in models that lack realism and predictive power. Therefore, an essential aspect of modelling the spatio-temporal evolution of IR lies in obtaining accurate and representative initial conditions, which often requires collecting baseline data and incorporating them into the modelling process. By doing so, models can better capture the actual dynamics of IR as it unfolds over time and space, leading to more reliable and insightful predictions.

## Conclusions

The reviewed studies have primarily focused on “mapping” the spatial trends of IR in malaria vectors to gain insights into the spatial and temporal distribution of IR within these vectors. A prevalent observation is the predominant use of static models for spatial IR modelling, with limited exploration of dynamic modelling approaches. Dynamic modelling approaches hold significant potential, particularly for capturing the temporal dynamics associated with the evolution of IR over time, as vectors adapt to the selective pressures exerted by insecticides. Additionally, we conclude that there is untapped potential to enhance the robustness of models employed for spatial and temporal IR modelling by incorporating additional IR drivers. Furthermore, a notable omission in the reviewed articles is the integration of thermodynamic and diffusion principles into the modelling framework. This remains an unexplored area that holds promise for further investigation. In essence, these conclusions underscore the need for continued research and innovation in the field of spatial and temporal modelling of IR in malaria vectors. A more comprehensive and dynamic modelling approach, coupled with the inclusion of additional drivers and the exploration of thermodynamic principles, can contribute to a deeper understanding of IR dynamics and lead to more effective strategies for its management and control. The practical outcomes of our review underscore the essential function of modelling in pinpointing locations affected by IR, mapping its proliferation and deciphering its evolution over time. The insights garnered from these models are pivotal in creating a solid base for devising effective intervention strategies and formulating recommendations that guide decision-making. Therefore, the intelligence acquired from modelling efforts plays a critical role in shaping actionable recommendations, significantly influencing the strategies employed in malaria vector control. This underscores the value of modelling not just as a theoretical exercise, but also as a practical tool with direct implications for public health and policy formulation.

### Supplementary Information

**Additional file 1: Table S1.** Summary of articles included in final systematic review follow the screening process.

**Additional file 2.**Summary on approaches used to model and map insecticide resistance.

## Data Availability

Not applicable.
