# Supplementary material for: Insights and challenges of insecticide resistance modelling in malaria vectors: a review
Source: Parasit Vectors. 2024 Apr 3;17:174. doi: 10.1186/s13071-024-06237-1 (PMC10993508; doi:10.1186/s13071-024-06237-1)
Supplement: Supplementary file 1 — Additional file 1: Table S1. Summary of articles included in final systematic review follow the screening process. [file 13071_2024_6237_MOESM1_ESM.docx]

# **Additional information**

**Additional file 1: Table S1.** Summary of articles included in final systematic review follow the screening process

| Authors, year | Aim | Methodology | Outcome |
| --- | --- | --- | --- |
| [52] | Gather data on *Anopheles. gambiae s.s*. and *An. arabiensis* frequencies in Mali and examine their correlation with climatic and environmental factors to create spatial distribution maps. | Apply Bayesian geostatistical logistic regression models, using various drivers: temperature (max and min), rainfall, proximity to water bodies, NDVI, SWS index, land use, agro-ecological zones, and malaria transmission suitability | Generated spatial maps indicating *An. arabiensis* concentration in drier savannah regions and *An. gambiae s.s*. preference for southern savannah and riverbanks. Findings revealed positive correlations between *An. arabiensis* proportion and NDVI, SWS index, and maximum temperature, but inverse relationships with minimum temperature and rainfall. Limitations include higher precision errors in less-surveyed zones. |
| [53] | Spatial and temporal variation in the knockdown resistance (kdr) allele L1014S in *An. gambiae s.s*. and phenotypic variability in susceptibility to insecticides in Western Kenya | Used multiple logistic regression to analyse he frequency of L1014S homozygotes in *An. gambiae s.s* where the log (odds of kdr L1014S homozygosity) was modeled as a function of year and location | Findings reveal kdr frequency in *An. gambiae s.s*. increased dramatically from 1996 - 2010, coinciding with the scale up of insecticide-treated nets. Bioassays demonstrated that *An. gambiae s.s*. had moderate phenotypic levels of resistance to DDT, permethrin and deltamethrin while *An. arabiensis* was susceptible to all insecticides tested. |
| [33] | Evaluated how temperature affect the knockdown and mortality of *An. stephensi* females following their exposure to pyrethroids (permethrin) and organophosphates (malathion) | Applied generalized linear models (GLM) with the binomial error distribution and logit link function to analyze data on the exposure effect. In case of over-dispersion, Quasi-binomial distribution was applied. | Lowering of exposure temperature from 26^0^ C reduced susceptibility of the female *An. stephensi* to the WHO resistance-discriminating concentration of malathion. The study revealed that survival of insect after exposure to insecticide permethrin depended on mosquitos’ thermal history. The study is presents the impact of thermal history of mosquitoes to their survival upon exposure to insecticides. |
| [54] | To develop IR Mapper, which is an online mapping tool. | The IR mapper was developed using ArcGIS. The data used in IR mapper, is on susceptibility and resistance mechanisms of *Anopheles* species, extracted from published peer reviewed literature. | Data trends reveal consistent increase in the prevalence and distribution of confirmed resistance in *An. gambiae* s.l. to pyrethroids, between 2001 and 2012. The data on resistance and susceptibility to pyrethroids and DDT was more abundant than data on carbamates and organophosphates. Metabolic resistance mechanisms were detected in western and eastern African populations and the two kdr mutations (L1014S and L1014F) were widespread. |
| [55] | To investigate the temporal trends of IR in field-caught *An. gambiae* mosquitoes across the Côte d’Ivoire’s ecological zones, for the period 1993 to 2014. | Mapped spatial and temporal trends of IR using GIS and the IR mapper tool. GLMs with a binary logistic link function were used to test the effect of year of sample, ecological zone and their interaction on bioassay mortalities. | Bioassay mortality declined overtime, for all insecticide classes. Further, a significant spatio-temporal variation with stronger declines being observed in the southern ecological zones for DDT and pyrethroids in the central zone, but apparently opposite effect for the carbamate and organophosphate. Limitations highlighted and which affect modelling include sample-site specific biases, and spatial temporal variations in investigation effort. |
| [56] | Investigate the habitat suitability of mosquitos prior to implementation of large-scale ITN interventions in Tanzania, and how distribution of ITN target the areas where mosquitoes thrived. | Species distribution models (SDM) were constructed for the occurrence of *Anopheles* mosquitos for the period between 1999 and 2003. A 2011-2012 layer of mosquito ITN ownership was created using georeferenced data across Tanzania for comparison. | Elevation, land cover, and human population distribution outperformed variants of temperature and NDVI in resulting SDM. Further, the spatial distribution of ITN ownership across Tanzania was near-random spatially. In addition, presence-only species observations assembled from an array of sources that differ in sampling effort and geographical focus could bias models toward areas with easier access. |
| [45] | Build a geostatistical framework for estimating the spatial and temporal distribution, as well as the abundance of primary vectors in Nagongera sub-county, eastern Uganda for the period from October 2011 to December 2015. | A Bayesian hierarchical generalised mixed model with spatial and temporal effects predicted maps of vector densities, informed by; climatic (rainfall and temperature), ecological (EVI), topography, proxy measure of urbanicity, euclidean distance of household to water sources, and household density. | Rainfall and EVI were important drivers of vector density and seasonality while increasing distance from water sources was associated with lower mosquito density. The study observes that a different model formulation can result to different spatial variations further adding that adult mosquito dispersal mechanisms has potential of altering their spatial and temporal distribution significantly. |
| [57] | Analyse the association between kdr prevalence and environmental conditions in different regions across China. | The trend surface of kdr allele frequency was created using inverse distance weighted (IDW) function in ArcGIS. The prediction model was build using a two-stage method, radial basis function (RBF) to determine variable importance, then multi-layer perceptron (MLP) for prediction, informed by climatic, topographical/ landscape and agricultural variables. | Established that long term intensive use of insecticides resulted development of IR. Environmental conditions affected mosquitoes response to insecticide and the selection of resistance mechanisms. Further, the number of crops in a year was the most important predictor for the kdr mutation rate. Topography, long-term mean climate and land cover all contributed to the kdr mutation rate. |
| [58] | Understand the effect of bendiocarb spraying on the frequency of kdr L1014S homozygotes (RR) (kdr homozygosity) while controlling for other factors that influence development and spread of resistance. | Applied a logistic regression model whose covariates were Bendiocarb coverage, annual rainfall, altitude, mosquito collection method, LLIN use, LLINs distributed in the previous 5 years, household use of agricultural pesticides, and malaria prevalence in children 2-9 years old. | This study suggests that using a carbamate insecticide for IRS in areas with high levels of pyrethroid resistance may reduce kdr frequencies in *An. gambiae s.s*. |
| [59] | Estimate the seasonal population size variations, survival and dispersal of male mosquitoes of the *An. gambiae* complex in Bana Village | Mark-release-recapture experiments were carried out two consecutive years, during wet and dry seasons. Recapture proportion, between experiments were separately compared using either proportion tests or binomial-family GLMs. Analysis of variance (ANOVA) with stepwise deletion testing was applied to assess the influence of season, capture method and release location on the net distance dispersed. | Mosquito dispersal ranged from 40 to 549 m over the seven days of each study and was not influenced by the season, but mainly by the release location, which explained more than 44% of the variance in net dispersal distance. |
| [60] | To define the geographical distributions of dominant malaria vector sibling species in Africa | Two datasets on occurrence of sibling species, one dataset retrieved from published literature and other sources, and the other dataset (background data) on all *Anopheles* surveys in the region were used to inform SDM. Boosted regression trees method were used to predict the relative probability of species occurrence at every 5 km × 5 km pixel. Model validation validated using test data, with metric being area under the curve data | SDM identified combinations of environmental variables that best distinguished areas supporting species presence from the range of environments sampled. The models estimated the relative probability of species presence at all locations within the species range. The results data provide good coverage of each species in areas with both strong and small seasonal variations. |
| [29] | Modelling the spatial variations in the presence and IR for malaria vectors in Lao | Spatial temporal models, build using GIS, involved three sub-models on; 1) probability of the presence of *Anopheles*; 2) insecticide presence probability based on suitable conditions for insecticide use; 3) estimating human vulnerability. | The findings revealed spatio-temporal distribution of environmental risk of *Anopheles* presence as well as potential emergence of IR and the risks of human exposure to the threats. The models were informed by environmental variables only and other potential variables were not used. Lack of records on insecticide use in agriculture was a challenge faced as well as lack of data on the specific insecticide used. |
| [10] | To establish the associated patterns of IR in field populations of malaria vectors across Africa | Bayesian geostatistical models were applied to quantify patterns of covariation in resistance phenotypes across different insecticides. | The prevalence of resistance related with different insecticide types over a large part of sub-Saharan Africa. Further, there were associations between the average prevalence of the resistance phenotype for DDT and pyrethroid insecticides and the frequency of voltage-gated sodium channel (Vgsc) mutations. On challenges, estimation of resistance is complicated by the sparsity of observations in field populations, variation in resistance over time and space at local and regional scales, and cross-resistance between different insecticide types. |
| [61] | Modelling the effect of vector resistance to control strategies. | A model mathematical captured the development of resistance as well as loss of resistance in mosquitoes and how these affect the progress in malaria control. Important thresholds were calculated from mathematical analysis and numerical results. | Results reveal the existence of the malaria free and endemic equilibria whose existence and stability depended on the control reproduction number, R_c_. The disease persist when the R_c_ > 1 and dies out when R_c_ < 1. |
| [62] | To determine the impact of IR in malaria vectors on the protective effectiveness of LLINs and IRS, and assessing trends in the IR status and underlying mechanisms in the main malaria vector species in response to different interventions. | Longitudinal monitoring of phenotypic resistance to pyrethroids was undertaken in 290 clusters across Benin, Cameroon, India, Kenya and Sudan. Mortality in response to pyrethroids in the major an. vectors in each location was recorded during consecutive years using standard WHO test procedures. Trends in mosquito mortality were examined using generalised linear mixed-effect models. | IR was detected in clusters in all countries across the study period. The highest mosquito mortality was consistently reported from India, in an area where ITNs had only recently been introduced. Substantial temporal and spatial variation was evident in mortality measures in all countries. Overall, a trend of decreasing mosquito mortality was recorded. Pyrethroid resistance increased over the study duration in four out of five countries. |
| [11] | Mapping the trends in insecticide resistance phenotypes in African malaria vectors, for the period 2005 to 2017. | Applied Bayesian geostatistical ensemble modelling approach. The modelling approach, involving using machine-learning methods (XGB, RF and BGAM models) to extract predictor variables with high prediction power from a total of 111 predictor variables, then using Gaussian process to model the spatio-temporal error covariance structure. | Generated fine-scale predictive maps of resistance phenotypes in *An. gambiae* complex across Africa. Spatial heterogeneity in sampling distribution resulted in confining the analysis to two separate geographic regions of sub-Saharan Africa. On challenges and limitations, the available susceptibility test results for insecticides from other classes other than pyrethroids are relatively low, and also susceptibility test data for some species like *An. funestus* are insufficient to support such species-specific geospatial analysis. Also, estimation of resistance is complicated by the sparsity and heterogeneous distribution of observations in field populations, variation in resistance over time and space at local and regional scales, and cross-resistance between different insecticide types. |
| [12] | Evaluating IR across Africa districts. | The model for generating district-level maps was geostatistical ensemble model. | District-level maps were generated for the probability that pyrethroid resistance in *An. gambiae s.l*. exceeds the WHO thresholds for susceptibility and confirmed resistance. In addition, the criteria for deployment of piperonyl butoxide-treated nets, that can could potentially mitigate against the effects of metabolic resistance to pyrethroids were mapped. |
| [63] | To understand the location, scale, and driving factors of insecticide resistance that can enhance the ability of vector control organizations to target populations effectively. | The study tested for associations between landscape, demographic, and insecticide-use factors using a beta regression modelling approach and evaluated the effect of spatial lag and spatial error terms on overall explanatory power of these models. | Vegetation density, distance from roads, and pyrethroid use by vector control districts were consistently significant predictors of kdr genotype frequency in the top-performing beta regression models. Spatial lags improved model fit and explanatory power of the models.  The study concludes that populations within 20 km of each other are likely to have similar IICC frequencies if the sites have similar landscape features. |
| [64] | Demonstrating host seeking-behaviour, in the presence of spatial repellent using machine learning simulations. | The flight behaviour of mosquitoes was simulated using the self-propelled particle (SPP) model. The simulated random walk scenarios were undertaken with insecticide susceptible/resistant mosquitoes against repellent alone and against repellent plus attractant to mimic a human host. | In the absence of an attractant or repellant, mosquitos fly randomly to anywhere in the cage, but they fly towards an attractant if it is present. In the presence of a repellant, the insecticide-susceptible mosquitoes moved to the furthest corner of the cage, away from the repellent release point and a high proportion of highly resistant vectors might reached the attractant release point earlier in the simulation. |
| [65] | Assessing the trends of an. malaria vector resistance to the four major insecticide classes focusing on the year 2011 to 2016; and generate predictive maps of IR for the year 2017. | Used generalized additive mixed models (GAMM) to establish the IR trends, for each class of insecticide, while the models’ likelihood was based on a binomial distribution and sites were included in the model as random effect. Predictor variables included population, crops area coverage, area covered by urban, irrigated cropland and distance from water. | Predictive maps of IR for 2017 were developed using the model. Limitations include high uncertainty in the estimated because of noise in the data arising from IR testing and heterogeneity of sampling sites. In addition, Comprehensive IR data by season or mosquito sex is scarce, resulting in knowledge gap. |
| [29] | Modelling spatio-temporal trends in the frequency of genetic mutations conferring insecticide target-site resistance in African mosquito malaria vector species, which included *An. gambiae, An. coluzzii, and An. arabiensis* . The focus was two target-site resistance mutations in the Vgsc gene, 995S and 995F, with the time being the period 2005–2017. | A spatiotemporal modelling approaches which applied involving machine learning methods and Bayesian modelling approach. Ninety-nine potential predictor variables were involved. A Bayesian multinomial logit regression was used as a meta model to combine predictions across the three machine learning models | The coverage of ITNs was an influential predictor of Vgsc allele frequencies, with modeled relationships between ITN coverage and allele frequencies varying across species and geographic regions. Variables describing solar radiation and humidity were the highest ranking in terms of their impact on predicted allele frequencies. The resulting predictive maps show how spatiotemporal trends in insecticide target-site resistance mechanisms in African *An. gambiae* vary across individual vector species and geographic regions. |
| [66] | Fine-scale spatial distribution of deltamethrin resistance and population structure of *An. funestus* and *An. arabiensis* populations in Southern Mozambique | CDC bottle bioassay and PCR assays were performed with *Anopheles* mosquitoes to determine phenotypic and molecular IR profiles, respectively. Microsatellite analysis was conducted on a subsample of mosquitoes to estimate genetic diversity and population structure. | The results obtained show high level of gene flow among the mosquito populations. The data indicate that CYP6P9a resistance markers do not capture all phenotypic variation in the area, but also that resistance genes of high impact are likely to easily spread in the area. The spreading as highlighted in this study could potentially be attributed to spread of mosquitoes. |

GLM = generalized linear models, NDVI = Normalized difference vegetation index, SWS = Soil Water Storage, DDT = Dichlorodiphenyltrichloroethane, WHO = world heath organization, IR = insecticide resistance, GIS = geographic information system, CDC = center for disease control, PCR = Polymerase Chain Reaction, ITN = Insecticide-treated bednet, GAMM = generalized additive mixed models, XGB = extreme gradient boosting, RF= random forest, GAM = generalized additive models , LLINs = long-lasting insecticidal nets, IRS = Indoor residual spraying
