# Supplementary material for: Insights and challenges of insecticide resistance modelling in malaria vectors: a review
Source: Parasit Vectors. 2024 Apr 3;17:174. doi: 10.1186/s13071-024-06237-1 (PMC10993508; doi:10.1186/s13071-024-06237-1)
Supplement: Supplementary file 2 — Additional file 2.Summary on approaches used to model and map insecticide resistance. [file 13071_2024_6237_MOESM2_ESM.docx]

**Additional file 2:** Summary on approaches used to model and map insecticide resistance

*Bayesian geostatistical models*

Bayesian geostatistical models have been used in mapping IR over space and time. The models are anchored on Bayesian theorem which involves drawing inference from posterior distribution, utilizing prior distribution and the likelihood. In other words, Bayesian approach begins by specifying the distribution over parameters to be estimated. The prior distribution reflects the known information without inference to the dataset on which the model is estimated. In Bayesian approach therefore, prior probabilities are used to inform the outcome, and then continual update of the probabilities when new evidence is received. The model is therefore defined as (Eqn 1);

| $Y_{k}\left( \boldsymbol{s}_{\boldsymbol{i}}\boldsymbol{,t} \right)= X_{k}^{T}(s_{i},t)\boldsymbol{\beta}_{\boldsymbol{k}}\boldsymbol{+}w_{k}\left( s_{i},t \right)+\varepsilon_{k}(s_{i},t)$ | (1) |
| --- | --- |

where $Y_{k}(s_{i}, t)$ is the proportion of mortality record for a bioassay using insecticide active ingredient k, and where the bioassay test was conducted on a sample collected from location $s_{i}$ at time t. **β**_k_ is the vector of the model coefficients. X_k_($s_{i}$, t) is the vector of covariates informing the model.$w\left( s_{i},t \right)$is a Gaussian process which is modelled by a spatio-temporal Gaussian Markov random field (GMRF). ε(s, t) is the Gaussian white noise where ε(s, t) ∼ N(0, $\sigma_{k}^{2}$ ). Since the prior distributions for the model parameters are required to account for the prior information, the vector of prior distributions for the parameters can be denoted as θ_k_ =[$\beta_{k}, w_{k}, \sigma_{k}^{2}$]. With the prior distributions and the likelihood, the posterior distributions of θ_k_ is then estimated aided by R packages such as R-INLA package.

*Generalized linear models (GLMs), generalized additive models (GAMs) and generalized additive mixed models (GAMMs)*

GLMs have been used in the modelling IR in various studies (Additional file 1: Table S1). GLMs generally extends the traditional linear regression model, for the cases where response variables have non-normal error distributions or heterogenous variance. Examples where such modelling approach can be employed include modelling presence or absence of insecticide resistance (IR). In this context, the relationship between the response variable and the predictor variables is modelled through a systematic component, which is a linear combination of the covariates, and a random component which describes the distribution of the response variable [67]. The random component specifies the conditional distribution of the outcome variable Y_i_ given the covariates in the model. The linear function of the covariates is defined as (Eqn 2);

| ${}_{i}=\alpha+\beta_{1}X_{i1}+..+\beta_{k}X_{ik}$ | (2) |
| --- | --- |

The linearizing link function denoted as g(.) transforms the expectations of the response variable E(Yi) = $\mu$ to k linear predictors and defined as (Eqn 3);

| $g\left( \mu_{i} \right)={}_{i}=\alpha+\beta_{1}X_{i1}+...+\beta_{k}X_{ik}$ | (3) |
| --- | --- |

This approach has been used in some of the reviewed articles to establish the relationship between the presence/absence of IR and covariates [33,53,55, 59]. On the other hand, GAMs and GAMMs were also employed by studies to model IR [65] . These models are extensions of GLM and [68], incorporating fixed and random effects, and smoothing functions such as splines.
